# Recommended Interventions to Improve Human Papillomavirus Vaccination Uptake among Adolescents: A Review of Quality Improvement Methodologies

**DOI:** 10.3390/vaccines11081390

**Published:** 2023-08-21

**Authors:** Karniza Khalid, Kun Yun Lee, Nur Farihan Mukhtar, Othman Warijo

**Affiliations:** 1 Specialized Diagnostic Centre, Institute for Medical Research, National Institutes of Health, Ministry of Health Malaysia, Kuala Lumpur 50588, Malaysia; 2 Institute for Health Management, National Institutes of Health, Ministry of Health Malaysia, Shah Alam 40170, Malaysia; dr.leekunyun@moh.gov.my; 3 Department of Obstetrics and Gynecology, Hospital Tuanku Fauziah, Ministry of Health Malaysia, Kangar 01000, Malaysia; nurfarihan83@gmail.com; 4 Kedah State Health Department, Ministry of Health Malaysia, Alor Setar 05400, Malaysia; othman.w@moh.gov.my

**Keywords:** quality improvement, systematic review, papillomavirus vaccine, vaccination coverage, adolescent

## Abstract

Background: Routine human papillomavirus (HPV) vaccine uptake continues to be suboptimal since its recommendation in 2006 for girls and in 2011 for boys. This paper aims to review published quality improvement (QI) methodologies on interventions to improve HPV vaccine uptake among adolescents. Methods: Science Direct and Scopus databases were searched for QI initiatives evaluating the effect of multimodal interventions to improve HPV vaccination rates (initiation and/or completion of series) among adolescents. Studies that included an outcome of interest among adolescents aged 10 to 18 years old were included. Two investigators worked independently to screen for potential articles and a designated investigator extracted data on study characteristics and evaluated the outcomes. Results: A preliminary search yielded a total of 523 articles and 13 were included in the final analysis. Common strategies were provider-specific (i.e., webinar, telementoring, train-the-trainer approach) and patient- and/or parent-specific interventions (i.e., reminder emails, phone calls and text messages, social events), with an emphasis on education and knowledge empowerment. System-level interventions such as policy changes and revised protocols were less commonly prescribed despite being associated with a more significant weight on the overall outcome. Conclusions: Creative, sustainable, and economical multilevel interventions that focus not only on provider training and public education but also incorporate local policies and system enhancements can substantially improve HPV vaccination coverage among adolescents.

## 1. Introduction

Human papillomavirus (HPV) vaccine administration is recommended for adolescents by the age of 11 or 12 years to protect them from cancer-causing infections and precancers caused by HPV infection. HPV vaccination has been shown to be an effective primary prevention method to reduce the associated healthcare costs [1] through a two-dose 6-monthly interval vaccination series for all children aged 11 to 12 years [2]. In addition, the 9-valent HPV vaccine is highly protective against most HPV-related cancers and genital warts that are associated with high morbidity and mortality [1,3].

In the United States (US), recent data on the coverage of at least one dose of HPV vaccine among adolescents aged 13 to 17 years showed an increase from 71.5% in 2019 to 75.1% in 2020 [4]. Despite this promising observation, the figure is still well below the Healthy People 2020 recommendation that 80% of adolescents receive two doses of the HPV vaccine within 6 to 12 months [1,5]. The Healthy People initiative is part of the US federal government’s disease prevention agenda towards a healthier nation in which several key domains were led by the US Centers for Disease Control and Prevention (CDC). The CDC has long been playing a pivotal role in supporting efforts by various organizations and institutions that aim at improving the uptake of HPV vaccination.

Globally, a multitude of quality improvement (QI) strategies has been devised to improve HPV vaccination rates: focusing on provider education on effective communication and HPV vaccine recommendations, prioritizing HPV vaccination along with other scheduled vaccinations, establishing strong partnerships between local healthcare facilities and departments, as well as developing and disseminating HPV-related resources for the public and providers [6]. In Malaysia for example, an HPV vaccination program targeting school-based 13-year-old girls was launched in August 2010 as part of the National Immunization Program. Within two years of its implementation, approximately 250,000 girls were successfully vaccinated, achieving the national target of at least 80% [7].

To date, the barriers to complete HPV vaccine administration among adolescents largely stem from a lack of knowledge or misinformation among the public. A study among 400 women aged 17 to 26 years old in Krakow, Poland showed that nearly 70% of the respondents were aware that HPV vaccination may help prevent cervical cancer and yet only 8.5% were vaccinated against HPV [8]. A similar observation was shared among 1210 young women from Greece that reported a relatively low HPV vaccination rate despite the high awareness of the vaccine’s ability to prevent HPV infection [9]. This signifies that awareness does not always lead to action. Therefore, a successful launch of HPV vaccination programs may be advantageous if certain specific components are in place to allow and encourage people to accept them. This could be in the form of a routine informational campaign that especially targets those uninformed, and in the form of easier access to vaccination, particularly in remote areas [10]. Misconceptions contribute to parental concerns regarding vaccine safety and efficacy and subsequently hesitations to receive the vaccination [5]. The literature also recognizes a perceived lack of necessity among parents or caregivers for HPV vaccination because their child is still young and not sexually active as a barrier. The HPV vaccine is commonly misconstrued as a clearance to engage in risky sexual activities, thus further impeding its acceptance among certain populations [11,12]. The evaluation of current practices is vital to formulate the best approach to overcome these barriers and misconceptions. An improved level of health literacy among the public can promote the uptake of HPV vaccination. In this study, we aim to review published quality improvement (QI) reports on the interventions to improve HPV vaccination rates among adolescents in healthcare settings.

## 2. Methods

### 2.1. Database Search

This review involved a database search using a pre-defined search strategy on the Scopus and Science Direct databases. The keywords ‘human papillomavirus’ OR ‘HPV’ AND ‘vaccination’ AND ‘quality improvement’ were used.

Eligible studies were included if they met the inclusion criteria, i.e., published QI reports aiming to boost the HPV vaccination rate among adolescents 10 to 18 years old, with the primary outcome analyzed statistically, published between 1 January 2019 and 25 March 2023. Review articles, letters to the editor, comments, and case reports were excluded. The references of the included articles were also checked to ensure inclusivity. Only articles published in the English language were included. If data of interest were unavailable for a particular study, the corresponding author was contacted for detailed information.

### 2.2. Data Collection

A single author (K.K.) independently collected and collated search results based on predefined search strategies. Pooled abstracts from the citations were combined using Mendeley Desktop Version 1.19.8 and independently examined by two authors for article selection (K.Y.L. and N.F.M.) for final analysis. Discrepancies were resolved by the third author (O.W.). Final articles included for analysis were reviewed, the information extracted, and analyzed by a single author whose primary interest lies in QI methodology (K.K.) using a designated data extraction sheet. The data included the name of the first author, publication year, QI target population, mode of QI initiative, and results (i.e., effect on HPV vaccination rate pre- and post-intervention). At the end of the data extraction, the two researchers (K.Y.L. and N.F.M.) reviewed all key data and qualitatively assessed article quality.

### 2.3. Primary Outcome

The primary study outcome was the overall HPV vaccination rate (not limited to either first injection or completed injection series). The secondary outcomes included the effect of specific intervention on any focus group (i.e., physician’s recommendation and delivery style, subjective evaluation from healthcare personnel, and patients/caregivers’ perspectives).

### 2.4. Study Registration

The study protocol was registered with the National Medical Research Register of the Ministry of Health Malaysia (NMRR ID-23-01062-XDU). The study was exempted from institutional review board based on the Malaysian National Institutes of Health Guidelines for Conducting Research in Ministry of Health (MOH) Institutions and Facilities 3rd ed. (2021).

## 3. Results

### 3.1. Search Results

**Figure 1:**
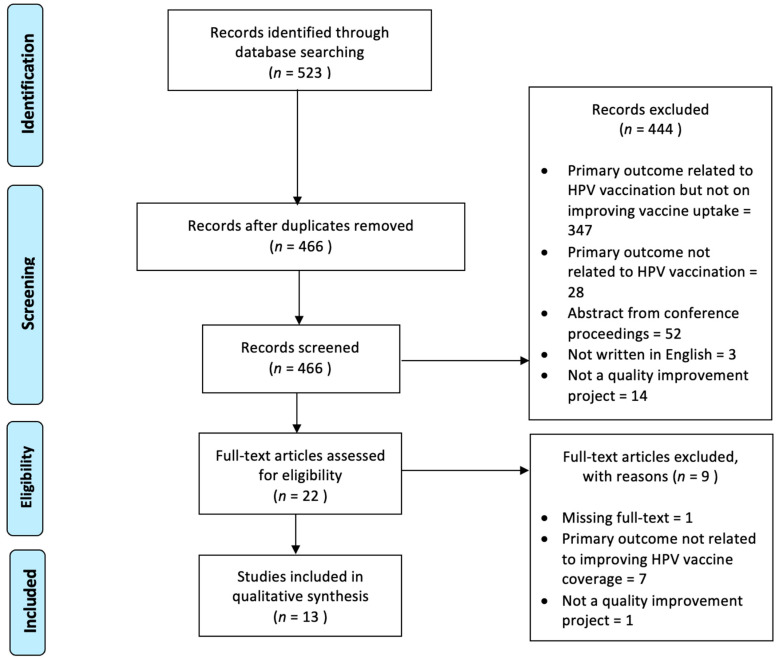
PRISMA flowchart for study selection. *n* = number of papers.

The article selection process followed the recommendation by the Preferred Reporting Items for Systematic Reviews and Meta-Analysis (PRISMA) guidelines (Figure 1).

Table 1 presents the main characteristics of the 13 QI reports included in the systematic review. Three key intervention targets emerged as the QI approach towards improving HPV vaccination rates among adolescents, i.e., reshaping the perspectives of the healthcare providers and of the adolescents and/or the caregivers, as well as the refinement of the local healthcare system.

### 3.2. Healthcare Provider-Targeted Empowerment Approach

From the papers included in this review, improving the knowledge and communication skills of healthcare providers (HCP) through education sessions was the most common QI initiative undertaken to empower the HCPs, and subsequently boost the HPV vaccination rate in healthcare settings [1,13,14,15,16,17,18,19,20,21,22,23]. The topics covered during these sessions encompassed the global and national burden of HPV-related diseases, an updated HPV vaccination schedule, and ways to successfully communicate with parents and adolescents about the HPV vaccine.

In addition, for the QI activities to be successful, HCPs should have access to reliable resources on ways to improve HPV vaccination among adolescents in the primary care settings. More importantly, these resources should be developed based on the recommendations of key stakeholders such as the CDC, the American Academy of Pediatrics (AAP), the Henry F. Kaiser Family Foundation, etc. [5]. Furthermore, all materials should be easily accessible through websites and prepared in interactive formats such as videos and articles. User-friendliness of these materials can enhance the providers’ knowledge on the HPV vaccination and aid in the discussion with parents and/or adolescents, as well as provide strategies to deal with difficult parents and/or adolescents [5].

As for the QI coaches, in addition to playing an important role in educating the participants, they should also be actively involved in the project to continually motivate and provide feedback about on-site performance to the team members. A dynamic team is vital to sustain the momentum and continuum of the QI project to achieve the desired objectives [5]. For example, a QI project involving 34 pediatricians designed an ‘ECHO’ program whereby HCPs convened in a monthly one-hour video conference with the QI project leaders for a total of nine months. The sessions started with a 15–20 min educational lesson covering topics related to HPV vaccination for the HCPs. The QI project leaders then reinforced the implementation of the evidence-based interventions. This was followed by interactive sessions involving simulations of patient encounters as well as sharing of experiences and ideas to boost HPV vaccination among the providers [19].

### 3.3. Reshaping Adolescents’, Parents’, and/or Caregivers’ Perception of HPV Vaccination

It is well established that risk communication and community engagement are fundamental to achieve high public acceptance and uptake of vaccinations. This can be achieved by empowering the public with adequate knowledge, awareness, and confidence through effective communication and strategic social media approaches.

In this review, one of the QI strategies employed to prime the parents and/or caregivers in preparation for their child’s vaccination was to send a pre-visit email with information about the HPV vaccine prior to their child’s scheduled visit [13,15]. Some QI initiatives even took a step further by discussing about HPV vaccination earlier on (by the age of 9) [22] and created a client reminder system through phone calls or text messages [17,21]. Another QI project established a recall system to trace patients who did not turn up to the vaccination appointment [18].

Furthermore, social events to improve awareness and health literacy among the public were also organized, for example the HPV vaccination poster contest and ‘HPV T-shirt Fridays’ [17]. By providing the right information to the public via reliable and easily accessible resources, the public can be assured of the safety and efficacy of the vaccine. This includes placing HPV vaccination-related posters in high traffic clinic areas as well as distributing HPV educational brochures to eligible patients during clinic visits [23].

### 3.4. Redesigning Healthcare Systems and Changing Policies

Last but not least, effective healthcare policy changes may also bolster public health initiatives in improving HPV vaccination uptake. For example, a QI project conducted in a private pediatric practice in suburban New England found that the inclusion of HPV vaccination along with other vaccines contributed to an increase in HPV vaccination rates among adolescents from 17.8% to 63.6% in three months [13]. Several other strategies included activating a licensed professional-initiated protocol (LPIP) (led by nursing staff) when an eligible patient for vaccination is scheduled for a visit [22], daily review of patients with incomplete vaccinations for them to be individually traced [17], and an electronic medical record to support the tracking of patients eligible for vaccination [23].

## 4. Discussion

This review summarizes various QI methodologies used as evidence-based strategies in enhancing the uptake of HPV vaccination among adolescents. While most of the QI projects have shown a certain level of success, the implementation of these strategies in the real-world practice required careful planning and execution to ensure the feasibility and impact of the strategies.

Based on this review, most of the implemented interventions to improve HPV vaccination uptake and completion focused on advancing HCPs’ knowledge so that they can educate the patients and parents/caregivers. Additionally, the important role of HCPs in providing effective communication is further highlighted in a study in which as many as 80% of the parents agreed that the provider’s recommendation during the clinic session was the main reason for them to approve their children’s vaccination [13]. Providers practicing a presumptive style over a conversational style were found to be associated with a higher rate of vaccine acceptance [1,17]. Hence, a standardized communication intervention offered as in-person sessions with free and readily available resources will enable the HCPs to successfully deliver consistent and reliable messages to patients. Improvement in vaccine uptake will only be evident once HCPs are able to communicate with their patients with confidence. Furthermore, specific considerations on the potential benefits from prophylactic anti-HPV vaccination should also be highlighted by healthcare professionals to parents and/or potential vaccine recipients to improve vaccination acceptance. This includes data on possible vaccine effectiveness in preventing malignant transformation, as well as accelerating disease clearance in already HPV-positive women. A cross-sectional survey among 1698 women proved that HPV vaccination significantly reduced the development of severe cervical precancers, and thereby the need for treatment, including their long-term related morbidity [24]. In another study, HPV vaccination was also found to be significantly associated with reduced HPV DNA positivity rates for the genotypes 16, 18, and 31 in women who were tested positive for HPV-16, prior to vaccination [25].

Despite HCPs being the most frequently targeted intervention group, a study conducted at three family practice clinics in Florida, US, to evaluate the use of a provider-based intervention (email, resource packets, education) showed only a slight improvement of HPV vaccination rates [5]. A successful plan–do–study–act (PDSA) process does not always equate to a successful QI program. Multiple intrinsic factors may potentially influence the overall outcome, such as poor resources, lack of teamwork and motivation among staff involved, inadequate organizational support, and interprofessional frictions (especially when involving various lines of work). Therefore, in addition to HCPs at the clinic level, change agents at multiple points in the wider healthcare systems also need to be involved for favorable decision-making and outcome of vaccination uptake.

Our study amplifies the significant role of social networking in healthcare. Provision of educational resources for patients/caregivers, regular reminders about clinic visits for scheduled vaccination via various available media, and awareness-raising campaigns and events were proven to be compelling efforts in boosting vaccination rates. Knowledge empowers adolescents (and their caregivers) to make proactive and appropriate decisions regarding the HPV vaccination. Hence, transparency in providing information regarding vaccine safety will also alleviate fears and misperceptions and guide their decision to get vaccinated [1,14]. Apart from that, patient empowerment can also be achieved by partnering with neighborhood community centers or regional foundations to organize health literacy programs [17]. Health empowerment results from an interplay of perceived information benefits and decision-making benefits, that is influenced by the perceived argument quality and the credibility of the source information. In the current digital and internet era, online health information providers should always pace up with the recent advances in medicine and current medical recommendations based on legitimate resources, strengthen information quality, and provide a differentiated level of information based on the audience level of understanding and health literacy [26].

In comparison, some of the less commonly implemented interventions often require systematic changes, such as the establishment of an integrated patient-tracing system and scheduled follow-ups after missed appointments, facilitated through electronic health records. These multi-mode reminders via regular mail, telephone calls, e-mails, and text messages can be used to reach the young adult population [18]. The same paper found that staff calls made to younger patients aged 11 to 17 years yielded up to 45% attendance for vaccination compared to just 6% among the 18–26-year-olds. Similarly, the distribution of educational brochures via mail followed by a telephone discussion was also less successful at increasing HPV vaccination rates among individuals 18 to 26 years old with incomplete vaccination [21]. Therefore, while more emphasis is given to infrastructures that can improve patient recall and tracking, they should be targeted at the right age group to optimize the outcomes [27,28].

Apart from the aforementioned strategies, a study found that including HPV vaccination together with other vaccinations during adolescence (meningococcal and Tdap) also improved the overall vaccination rate [20]. This would not only simplify the process in achieving the desired vaccination rate but will also reduce the need for multiple clinic visits. On another note, Singh (2022) reported that most of the HPV vaccination was performed during acute appointments rather than scheduled ones [21]. Hence, a system alert to trigger the arrival of any eligible patients for any type of consultations in the clinic will be beneficial to avoid missed opportunities. Therefore, the clinic receptionist should be attentive when registering patients to alert HCPs to potential vaccine recipients with a special tag on the medical record.

Apart from these evidence-based QI approaches, potential cultural factors may also influence the decision to vaccinate. A relevant study across 12 clinics in Ohio, US, found that Latino children recorded the highest HPV vaccination completion rate (80%) while White children showed the lowest rate at 64% [22]. However, any observed ethnic disparity in study outcomes should be interpreted with caution as it might be affected by the demographic distribution of the study population of interest because the income status of the neighborhood and the proportion of publicly insured patients would affect the access to medical treatment [29,30]. Therefore, when designing and implementing any QI strategies, local cultural influences must be taken into account to ensure the highest program outreach, especially in multiethnic, multicultural, and multilingual regions.

It is important to highlight the healthcare cost burden of preventable diseases caused by human papillomavirus. A recent review paper by Chesson et al. (2019) applying the current cancer cost estimates noted a substantial impact on the estimated medical costs that may be averted by HPV vaccination, with a moderate influence on the estimated cost per quality-adjusted life year (QALY) from HPV vaccination. In their example, a catch-up vaccination for adolescents and young adults was able to reduce the estimated cost per QALY by about USD 12,400 [31]. This further supports the initiatives to boost HPV vaccination programs, especially in areas with high burden of cervical cancer, low-resource settings, and scarce screening opportunities [32].

The findings from our review highlighted that QI initiatives for the improvement of HPV vaccine uptake among adolescents require holistic efforts that incorporate improvement in any given measure to overcome conventional practices [17]. Due attention ought to be paid to capture updates on the process and methods of improvements, as well as define individual roles and learnings points from the project. The procedures and practices developed during the QI project should also be integrated as part of the institutional practice to ensure sustainability. Lastly, we strongly encourage future QI reports to adhere to the SQUIRE guidelines in an effort to strengthen the reporting evidence for improvement in healthcare [33].

## 5. Conclusions

Multilevel interventions that focus not only on provider training, but also incorporates local policy and system refinements can substantially improve HPV vaccination uptake. Provider-based QI interventions are instrumental in improving the communication between clinicians and their patients with the aim of straightening HPV vaccine awareness and uptake. However, the implementation of these QI strategies in the real world can be challenging due to limited resources and demanding commitment. Therefore, a consistent and continuous HPV vaccination communication, supplemented by low-cost sensory rewards, as well as dynamic engagement between clinicians, supporting clinical staff, and the community are needed to ensure a high HPV vaccination uptake and completion among adolescents and young adults.

## Figures and Tables

**Table 1:** Summary of studies included in the final analysis.

No.	First Author, Year	Intervention Focus Group	Mode of Intervention	Primary Outcome	Results
1	Berstein, 2022 [13]	System/policy	Inclusion of HPV vaccination with other scheduled vaccines for adolescents	HPV vaccines rates among 11–12 year-old teens	HPV vaccination rate increased from 17.8% to 63.6%
		Patients	Parents were sent a pre-visit email containing vaccine information prior to annual healthcare visit		
		HCP	Initiatives to improve provider communication skills		
2	Davis, 2022 [1]	HCP	Educational intervention	HPV vaccination rates in children 11−12 years old	The quality of vaccine recommendations was improved Providers’ delivery approach via presumptive vs. conversational style increases vaccination rates
3	Gilkey, 2019 [14]	HCP	An in-clinic training session (includes instructions and a video on effective communication)	HPV vaccination coverage among patients aged 12 to 14 years	HPV vaccination coverage increased in the QI arm by 8.6%
4	Mackey, 2019 [15]	HCP	Reminder system for both physicians and patients	HPV vaccination coverage among patients aged 11 to 12	The immunization rate (for the first injection) was significantly greater than baseline
		Patients	Reminder mails to caregivers of patients aged 11–12 years		
5	Mathur, 2019 [16]	HCP	Four 1 h webinars highlighting on effective communication with parents regarding the HPV vaccine	HPV vaccination coverage (dose 1) for all 11- and 12-year-olds	Initiation of HPV vaccination increased in all participating practices (from 56.4% to 71.2%)
6	McGaffey, 2019 [17]	HCP	Periodic training on presumptive approach in HPV vaccine recommendation	HPV vaccination rate among 9–26 year-old patients (both male and female)	HPV vaccine initiation significantly increased by 12.8 PP in males and 10.6 PP in females Higher HPV vaccine completion in males by 16 PP and 10.9 PP in females
		Patients	Poster contest with HPV vaccine theme HPV seasonal posters displayed in high traffic areas Educational materials to parents and patients at appropriate health literacy level Review records to identify eligible patients who were unvaccinated or incompletely vaccinated Social reminder via phone calls or text messages for dose 2 and dose 3 Incentives for receiving HPV vaccination HPV T-shirt Fridays for social networking and awareness Parents to sign consent for dose 2 and 3 at the time dose 1 was administered		
		System/policy	Specific local protocol for HPV vaccination Review of patients with incomplete vaccinations		
7	Nissen, 2019 [18]	Patients	Implement reminder and recall system for eligible patients who were unvaccinated or with incomplete vaccination	The rate of HPV vaccine administration	In patients ages 11–26 years, HPV vaccine completion rates increased by 13% Zero-dose HPV vaccination dropped by 22% in seven pilot clinics over the two-year interventional period
		HCP	Vaccine education Provider assessment and feedback system Re-education on standing protocol orders		
8	Oliver, 2020 [19]	HCP	Telementoring platform with video conferencing to educate HCPs through case-based learning and brief lectures on various health conditions	Impact on HPV immunization rates (HPV initiation, series completion, and missed opportunities)	Self-reported increased confidence among the physicians when communicating with vaccine-hesitant families No improvement was observed for HPV vaccine initiation or series completion
9	Perkins, 2021 [20]	HCP	Train-the-trainer approach conducted in a 2.5 day conference	Vaccination rates among adolescents who turned 13 years	Series initiation increased by 23.6 PP (from 47.2 to 70.8%) HPV completion rates increased by 22.7 PP (from 24.6 to 46.3%). 90% of clinicians reported positive changes in their patient care and consultations based on what they had learned
10	Singh, 2022 [21]	Patients	Eligible patients received HPV educational brochures and CDC HPV vaccine information via mail or during in-person appointments (acute/scheduled visits). A follow-up telephone call was given around 2 weeks after the education brochure was distributed	HPV vaccination rates among young adults	The vaccination rate among patients who were seen in person at the clinic improved from 2.5% (1 of 40) to 46.6% (21 of 45).
		HCP	Clinicians and staff were briefed on the background and significance of HPV vaccination among teens, the current state of the problem, and the HPV vaccine guidelines over a video conference All HCP members were involved in the education (including physicians, medical assistants, nurses, and supporting staffs) An audit and a feedback method were used to keep staff engaged in the QI project		
11	Smajlovic, 2023 [22]	Patients	Announcement approach–HCP discusses the need for the HPV vaccination from the age of 9 years with the parent and child CDC posters on the importance of HPV vaccination in the prevention of various cancers and educational handouts were strategically placed in consulting rooms/clinics An information sheet on the rationale and risks/benefits of the HPV vaccine	HPV-vaccination series completion rate to 70% among the 13-year-old patient population	The overall HPV vaccination series completion rates increased from 27% to 65%
		HCP	QI team provided feedback via email to providers on a 6-monthly to a yearly basis on the monitored HPV vaccine completion rates among 13-year-old adolescents		
		System	A licensed professional-initiated protocol (LPIP) dedicated for nursing staff is triggered when an eligible patient is scheduled for a visit		
12	Wallace-Brodeur, 2020 [3]	HCP	Training sessions on effective recommendation for HPV vaccine, strategies to reduce Mos, and techniques on motivational interviewing	Reduction in Mos (defined as any visit that a patient did not receive the vaccination dose despite being eligible)	LHDs reduced MOs for HPV vaccination by 25.3%
13	Zorn, 2023 [23]	HCP	Provider and staff training, with peer-to-peer coaching by QI champion	HPV initiation at age 9 and series completion rates	HPV initiation rates increased by at least 30% in the first year of intervention Sustained improvements in initiation and series completion at age 11–12 years by up to 40%
		Setting/system	Electronic medical records support		
		Patients	Printed educational resources Immunization schedule posters in examination rooms		

Abbreviations: ACS = American Cancer Society; CDC = Center for Disease Control; HCP = healthcare professional; HPV = human papillomavirus; LHDs = local health departments; MO = missed opportunity; PP = percentage points; QI = quality improvement.

## Data Availability

The authors confirm that the data supporting the findings of this study are available within the article.
